# Monocytes/macrophages express chemokine receptor CCR9 in rheumatoid arthritis and CCL25 stimulates their differentiation

**DOI:** 10.1186/ar3120

**Published:** 2010-08-25

**Authors:** Caroline Schmutz, Alison Cartwright, Helen Williams, Oliver Haworth, John HH Williams, Andrew Filer, Mike Salmon, Christopher D Buckley, Jim Middleton

**Affiliations:** 1 Leopold Muller Arthritis Research Centre, Medical School, Keele University, RJAH Orthopaedic Hospital, Oswestry, Shropshire, SY10 7AG, UK; 2 Rheumatology Research Group, MRC Centre for Immune Regulation, Institute for Biomedical Research, University of Birmingham, Vincent Drive, Edgbaston, Birmingham, B15 2TT, UK; 3 Chester Centre for Stress Research, University of Chester, Parkgate Road, Chester CH1 4BJ, UK; 4 Dept of Oral and Dental Science, Faculty of Medicine and Dentistry, Lower Maudlin Street, University of Bristol, BS1 2LY, UK

## Abstract

**Introduction:**

Monocytes/macrophages accumulate in the rheumatoid (RA) synovium where they play a central role in inflammation and joint destruction. Identification of molecules involved in their accumulation and differentiation is important to inform therapeutic strategies. This study investigated the expression and function of chemokine receptor CCR9 in the peripheral blood (PB) and synovium of RA, non-RA patients and healthy volunteers.

**Methods:**

CCR9 expression on PB monocytes/macrophages was analysed by flow cytometry and in synovium by immunofluorescence. Chemokine receptor CCR9 mRNA expression was examined in RA and non-RA synovium, monocytes/macrophages from PB and synovial fluid (SF) of RA patients and PB of healthy donors using the reverse transcription polymerase chain reaction (RT-PCR). Monocyte differentiation and chemotaxis to chemokine ligand 25 (CCL25)/TECK were used to study CCR9 function.

**Results:**

CCR9 was expressed by PB monocytes/macrophages in RA and healthy donors, and increased in RA. In RA and non-RA synovia, CCR9 co-localised with cluster of differentiation 14+ (CD14+) and cluster of differentiation 68+ (CD68+) macrophages, and was more abundant in RA synovium. CCR9 mRNA was detected in the synovia of all RA patients and in some non-RA controls, and monocytes/macrophages from PB and SF of RA and healthy controls. CCL25 was detected in RA and non-RA synovia where it co-localised with CD14+ and CD68+ cells. Tumour necrosis factor alpha (TNFα) increased CCR9 expression on human acute monocytic leukemia cell line THP-1 monocytic cells. CCL25 induced a stronger monocyte differentiation in RA compared to healthy donors. CCL25 induced significant chemotaxis of PB monocytes but not consistently among individuals.

**Conclusions:**

CCR9 expression by monocytes is increased in RA. CCL25 may be involved in the differentiation of monocytes to macrophages particularly in RA.

## Introduction

Rheumatoid arthritis (RA) is a chronic inflammatory disease resulting in the accumulation of macrophages, T cells and B cells within the synovium. The accumulation of these cells is involved in the development of inflammation, joint destruction and pain [1]. Monocytes migrate from the blood across the walls of synovial blood vessels and differentiate into macrophages. The clinical importance of monocytes/macrophages is revealed by the correlation between their number, disease activity and radiographic progression [2-4] and by the beneficial effect of therapies that target these cells (such as gold salts [5], methotrexate [6]) and monocyte depletion [7,8]. Furthermore, monocytes/macrophages are a major source of TNFα in the RA joint and blocking this cytokine has had a major impact on RA therapy [9,10].

Leukocyte recruitment is dependent on a cascade of events mediated by chemokines, chemokine receptors and adhesion molecules [11,12]. The homing, recruiting, organising and retaining activities of chemokines is dependent on the presence of and interaction with chemokine receptors on the leukocyte surface. *In vitro *and *in vivo *experiments have suggested that disrupting this interaction could potentially reduce inflammation and therefore offer a potential therapeutic approach [11]. Evidence has accumulated to show that chemokine receptors and their ligands are involved in the migration and retention of leukocytes in the RA joint [13,14]. For example CCR1, 2, 3 and 5 and CXCR4 are suggested to be involved for monocytes and CCR5, CXCR3 and CXCR4 for T lymphocytes.

CCR9 is reported as a receptor on lymphoid cells and is expressed both constitutively and in inflammation. It occurs on T lymphocytes of the small intestine, thymus, lymph node and spleen [15-17]. CCR9 is involved in T cell homing to the small intestine [15], T cell development and migration in the thymus [15,18,19], and recruitment of T cells in chronic inflammatory disease [20], being a target in inflammatory bowel disease. Finally it has been suggested that CCR9 is implicated in the survival or proliferation of T cell acute lymphoblastic leukemia cells [21]. Therefore CCR9 has mainly been reported to function in lymphocyte migration although other cell types, such as tumour cells, can express CCR9, playing a role in prostate cancer cell migration and invasion [22].

Contrary to many other chemokine receptors CCR9 binds only one known ligand, CCL25/TECK. This chemokine was originally defined as being chemotactic for activated, but not resting, human monocytic cells and activated mouse peritoneal macrophages [23]. This implies the existence of CCR9 on activated monocytes and macrophages; however, there has been a lack of studies of CCR9 on these cells in humans and mice. Monocyte/macrophage activation and differentiation play an important role in RA pathogenesis and their recruitment to RA joints is a central feature of the disease. Therefore we examined the expression and function of CCR9 by monocytes and macrophages in RA.

## Materials and methods

### Patient and control samples

RA patients (*n *= 24) were aged 33 to 91 (19 female, 5 male), had disease duration of 1 to 42 years, active disease defined by elevated ESR and CRP, positive rheumatoid factor (apart from three patients), and took disease-modifying drugs (etanercept, methotrexate, leflunomide or hydroxychloroquine) or steroids. The non-RA subjects (*n *= 7) were aged 42 to 60 years (six male, one female), had no history of RA and were medication-free [24]. Normal healthy volunteers (*n *= 20) were aged 24 to 63 (11 female, 9 male), had no joint symptoms and were medication-free.

The synovial samples were obtained from RA and non-RA patients following total knee replacement and arthroscopy respectively. RA synovia showed classic synovitis with lymphocyte and macrophage infiltration of the sublining, and lining layer thickening (as previously described [24,25]); with little or no fibrosis. Non-RA synovia were from knees with suspected meniscal or cartilage damage. Biopsies were from arthroscopically non-inflamed sites and subsequent histology showed they were essentially normal with no post-traumatic synovitis [24,25].

RA patients included in this study fulfilled the American Rheumatism Association criteria for RA. All samples were taken with informed consent and ethical approval.

### Flow cytometry

RA peripheral blood (PB) and matching synovial fluid (SF) and healthy PB were collected into preservative-free heparin. SF was treated with hyaluronidase for 30 minutes at 37°C. PB and SF mononuclear cells were isolated by gradient centrifugation with Ficoll-Paque Plus (Amersham-Biosciences, Little Chalfont, UK). Analysis of chemokine receptor surface expression was performed using three-colour immunofluorescence, as previously described [26]. After blocking non-specific binding with 10% IV-Ig (Flebogamma, Grifols, Cambridge, UK Ltd), PB and SF mononuclear cells (3 × 10^5^) were stained with anti-chemokine receptor antibody or isotype control, followed by FITC-labelled secondary antibodies (Cambridge Biosciences, Cambridge, UK). After blocking non-specific binding sites with mouse serum, cells were further stained with directly conjugated CD14-PECy5 and CD16-PE antibodies. The samples were analysed on an EPICS XL flow cytometer (Beckman Coulter, High Wycombe, UK). Cytometer calibration was standardized using fluorospheres (Flow-Set, Coulter). Data were analysed using WinMDI version 2.8 (The Scripps Institute, La Jolla, CA, USA).

### Reverse transcription-polymerase chain reaction (RT-PCR)

Total RNA extraction and RT-PCR was as our previous study [24] except that specific primers (MWG Biotech, Ebersberg, Germany) for *CCR9 *(product size 572 bp, [Genbank:NM_031200]), *CCL25 *(product size 311 bp, [GenBank:NM_005624]) and *L27 *(product size 344 bp, [GenBank:BC007273]) were used. The RT-PCR conditions were one cycle at (94°C for three minutes, 57°C for one minute, 72°C for one minute), X cycles at (94°C for one minute, 57°C for one minute, 72°C for one minute) and one cycle at (94°C for one minute, 57°C for one minute, 72°C for 10 minutes). X equals 34 cycles for *CCR9*, 35 cycles for *CCL25 *and 24 cycles for *L27*.

### Immunohistology

Immunohistochemistry using paraffin embedded sections were as [24] except that antigen demasking was for 15 minutes microwaving in citrate buffer (pH 6.0). H_2_O_2 _treatment was not necessary. Non-specific sites were blocked with normal horse serum (in phosphate buffered saline (PBS)) for 10 minutes before applying anti-human CCR9 goat polyclonal antibody (1:250, Capralogics, Hardwick, MA, USA) and the respective IgG control (Dako, Ely, UK). Antibody detection was by the Vectastain ABC Elite kit (Vector, Peterborough, UK) and 3,3'-diaminobenzidine (DAB) (Vector). Sections were rinsed and counter stained in Mayer's haematoxylin.

For double label immunofluorescence, cryosections (6 μm thick) were fixed in acetone for 10 minutes on ice, rinsed with PBS and incubated for one hour with CCR9 goat polyclonal antibody (as above 1:250) together with antibodies to either CD14 (1:20), CD68 (1:50), CD20 (1:100) or CD3 (1:50) (all from Dako, Ely, UK). Detection of CCR9 was with donkey anti-goat Alexa 594 and the other primary antibodies with goat anti-mouse Alexa 488 secondary antibodies, containing 10% human serum. For CCL25 the slides were incubated with anti-human CCL25 mouse monoclonal antibody (1:200, R&D Systems, Abingdon, UK) together with anti-CD14 or -CD68. CCL25 was then detected with isotype specific goat anti-mouse IgG2b Alexa 488, and CD68 and CD14 were detected with goat anti-mouse IgG1 and IgG2a Alexa 594 second antibodies respectively. Finally nuclear staining was performed with DAPI for three minutes, before mounting.

Controls were using species- or isotype-matched IgGs (Dako) instead of primary antibodies followed by the respective Alexa-labelled secondary antibodies.

### Stimulation of THP-1 cells with TNFα

THP-1 cells were grown in RPMI medium with 10% FBS and treated with TNFα (0 to 500 ng/ml) (PeproTech, London, UK) for 16 hours. They were then incubated with anti-CCR9 mouse monoclonal antibody (1:10; R&D Systems) followed by goat anti-mouse IgG2a-RPE conjugate (1:100, Southern Biotech, Birmingham, AL, USA) and analysed with a Becton Dickinson FACSCAN.

### Monocyte differentiation

PB was taken and erythrocytes eliminated using lysing buffer. Leukocytes (3 × 10^5 ^cells/well) were treated with hrCCL25 (0 to 500 ng/ml) (Peprotech UK), hrCXCL16 (0 to 500 ng/ml) (Peprotech, UK) or phorbal myristate acetate (PMA) (10 ng/ml; Sigma, Poole, UK) in RPMI containing 10% heat inactivated FBS for three hours at 37°C. They were incubated with CD14PE (1:50 in PBS with 5% FBS; BD Biosciences, Oxford, UK) and CD36-FITC antibodies (1:250 in PBS with 5% FBS; Santa Cruz Biotechnology, Santa Cruz, CA, USA) for 30 minutes (4°C). Monocyte populations were gated using FSC/SSC and anti-CD14PE, and CD36 MFI determined using a BD FACS Canto Flow Cytometer.

From two of the RA and healthy subjects, PB monocytes were also isolated using CD14+ positive selection using MACS microbeads (Miltenyi Biotec, Bisley, UK) and treated as above. The stimulatory effects of CCL25 on CD36 expression were significant and similar to those obtained using the erythrocyte lysis method of leukocyte isolation (described above).

### Chemotaxis assay

PB monocytes were isolated by positive selection using CD14+ MACS microbeads (Miltenyi Biotec) according to the manufacturer's instructions. The migration of the CD14+ cells was assayed in Transwell (Corning Incorporated Costar, Cambridge, MA, USA) with 5 μm pore size filter. The bottom of the well was siliconised (Sigmacote, Sigma, UK) to prevent cell adhesion. Medium containing 0.5% FractionV BSA and 0 to 1,000 ng/ml of CCL25 or 100 ng/ml MCP-1 was added to the bottom chamber, the filters were dropped in the medium and 0.5 × 10^6 ^cells were immediately added. The transwells were incubated at 37°C for one hour and finally the cells in the upper and lower compartment were collected, stained with CD14 and CD16, and analysed by flow cytometry using an EPICS XL flow cytometer (as described above for flow cytometry).

### Statistics

T-tests were used to compare CCR9 expression by RA and normal monocytes using flow cytometry (significance level <0.05). The effect of TNFα concentrations on CCR9 expression was determined by ANOVA and then pair-wise comparisons by Dunnett's test. The influence of CCL25 and PMA on CD36 expression was analysed using multilevel or hierarchical regression analysis on log transformed data, using the ratio test to compare the various multilevel models and the Wald test to obtain *P-*values [27,28].

For quantitation of the number of CCR9/CD14 positive cells by immunofluorescence microscopy, five fields of view of the synovial sublining at ×600 magnification were examined per patient and the number of CCR9+, CD14+ and CCR9+/CD14+ cells counted. Thus the mean numbers of positive cells (± standard error) per field of view were obtained; the total number of cells sampled per field of view was assessed by counting the numbers of nuclei stained by DAPI. T tests were used to compare RA and non-RA data.

## Results

### CCR9 expression by PB monocytes

CCR9 expression by monocytes was examined from RA PB and healthy PB using flow cytometry. The total monocyte population and two subpopulations were defined according to their FSC/SSC characteristics and surface marker expression with total monocytes all being CD14+, and the two subpopulations being CD14+ and CD16- or CD14+ and CD16+. In accordance with previous results [29] the CD14+/CD16- and CD14+/CD16+ subpopulations were both present in all RA (*n *= 7) and healthy (*n *= 6) PB samples, however marked heterogeneity was observed in the RA SF, with these two CD14/CD16 subpopulations being observed together in only one out of seven SF samples analysed (data not shown).

**Figure 1:**
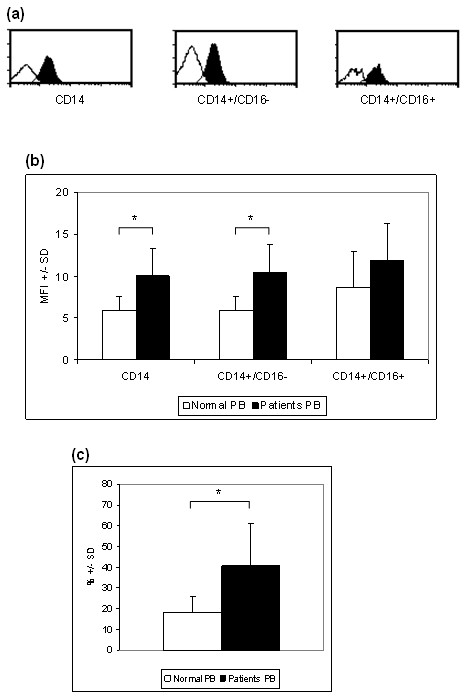
**CCR9 expression by monocytes in RA using flow cytometry**. **(a) **Representative histograms showing CCR9 expression by the total CD14+ population, and two subpopulations CD14+/CD16- and CD14+/CD16+ (solid fill), empty fill histograms represent the isotype-matched control used instead of anti-CCR9. **(b) **Results expressed as MFI (mean fluorescence intensity) showing CCR9 expression by monocytes from normal and rheumatoid peripheral blood (PB). **(c) **Results shown as percentage of monocytes expressing CCR9 in the total CD14+ population. * = significant difference between normal and rheumatoid PB (*P *< 0.05).

CCR9 was consistently observed in the CD14+ monocyte population and in the two subpopulations in both RA and healthy samples, examples of RA CCR9 and isotype control plots are shown in Figure 1a. CCR9 was significantly more abundant in RA PB than in normal PB in the CD14+ population and the CD14+/CD16- subpopulation (mean fluorescence intensity (MFI), *P *< 0.05) (Figure 1b). No significant difference was observed between the RA and normal CD14+/CD16+ subpopulation.

To determine if the number of CCR9 positive cells also varied between RA and healthy samples the percentage of CCR9+ monocytes was determined for the total CD14+ population. There were significantly more CCR9 positive CD14 monocytes in RA PB than normal PB (*P *< 0.05) above background (isotype control IgG2a) (Figure 1c).

The levels of CCR9 on CD14+ monocytes in the same RA PB and healthy PB samples were compared with other chemokine receptors. For RA PB the MFI (mean ± SD) for CCR1, CCR2, CCR5 and CXCR4 was 46 ± 13, 92 ± 20, 14 ± 10 and 17 ± 6, and for healthy PB 41 ± 17, 94 ± 40, 10 ± 4 and 11 ± 8 respectively. For RA PB the percentage positive cells (mean ± SD) for CCR1, CCR2, CCR5 and CXCR4 was 94 ± 10, 94 ± 8, 55 ± 31, 72 ± 13, and for healthy PB 99 ± 1, 99 ± 1, 58 ± 22 and 46 ± 26 respectively.

### CCR9 expression in synovium

**Figure 2:**
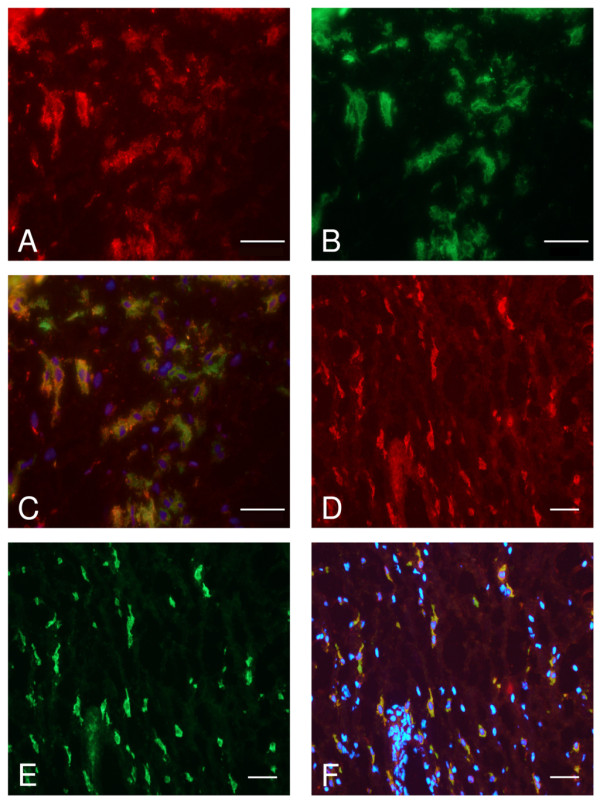
**Expression of CCR9 by macrophages in rheumatoid synovium**. Cryosections were treated with antibodies to CCR9 and CD14 or CD68 as markers of macrophages and double label immunofluorescence microscopy performed. **(a) **Shows staining for CCR9 (red) and **(b) **shows the same area stained for CD14 (green). **(c) **Shows a merge of images (a) and (b) and cells with colocalised CCR9 and CD14 (yellow). **(d, e) **Demonstrate staining for CCR9 and CD68 respectively and **(f) **is a merge of these two images, cells in yellow express CCR9 and CD68. Cell nuclei stain (blue) with DAPI in (c) and (f). The bar represents 50 μm.

CCR9 expression was examined by immunohistochemistry on paraffin embedded synovial sections. The receptor was detected in the synovia of all RA and non-RA patients, occurring on isolated cells in the tissue sublining at the edge of, or remote from, lymphoid follicles (data not shown). From their distribution and histological shape, these CCR9+ cells were identified as being potentially macrophages. To investigate this, double label immunofluorescence was performed on synovial cryosections using antibodies to CCR9 and markers of monocytes/macrophages. CCR9 co-localised with CD14 (Figure 2a-c) and CD68 (Figure 2d-f) in RA and non-RA synovia. This co-localisation occurred in the sublining using CD14 and CD68 markers and in some regions of the lining layer with the CD68 marker. No co-localisation was observed between CCR9 and CD20 (B lymphocytes) or CD3 (T lymphocytes) respectively in either RA or non-RA synovia (data not shown). Non-RA synovial samples displayed limited staining with CD3 and only occasional staining with CD20. No staining was detected using the isotype controls for all antibodies studied.

In order to quantitate differences in CCR9 expression by macrophages in RA and non-RA synovia, the number of CCR9+, CD14+ cells and CCR9+/CD14+ cells were counted in the sublining layer (Table 1). The total number of CCR9+ cells increased in RA compared to non-RA ST by approximately five-fold (from 12 to 63 cells per field of view, *P *< 0.001). As expected the number of CD14+ cells in control synovium was low, increasing 12-fold in RA (from 6 to 70 cells per field of view). Interestingly there was a large increase in the number of CCR9/CD14 double positive cells in RA compared to non-RA, amounting to 14-fold (from 4 to 57 cells per field of view, *P *< 0.004). The percentage of CD14+ macrophages that were positive for CCR9 was higher in RA than in non-RA synovia, being 81 and 66% respectively (as calculated by number of CCR9+/CD14+ cells ÷ number of CD14+ cells × 100 in Table 1). In addition, the vast majority of CCR9+ cells were CD14+ macrophages in RA synovium since 90% of these cells were CD14+ (as calculated by number of CCR9+/CD14+ ÷ CCR9+ cells × 100 in Table 1), whereas this figure was 33% in non-RA synovium.

**Table 1:** The number of double positive CCR9/CD14 cells in rheumatoid and non-rheumatoid synovial tissue

Patient	Number of CCR9+ cells (mean ± SE)	Number of CD14+ cells (mean ± SE)	Number of CCR9+/CD14+ cells (mean ± SE)
**Rheumatoid**			

1 (*n *= 92)	25 ±10	36 ± 9	25 ± 10
2 (*n *= 91)	59 ± 23	45 ± 25	43 ± 24
3 (*n *= 137)	64 ± 20	64 ± 20	51 ±12
4 (*n *= 136)	60 ± 10	60 ± 13	59 ± 12
5 (*n *= 173)	65 ± 23	80 ± 35	62 ± 24
6 (*n *= 166)	106 ± 9	135 ± 11	104 ± 9
**Overall mean ± SE**	**63 ± 10**	**70 ± 14**	**57 ± 11**

**Non-rheumatoid**			

1 (*n *= 76)	38 ± 12	1 ± 1	1 ± 1
2 (*n *= 77)	5 ± 5	4 ± 3	3 ± 3
3 (*n *= 70)	9 ± 4	4 ± 6	4 ± 6
4 (*n *= 47)	5 ± 1	5 ± 2	4 ± 3
5 (*n *= 75)	5 ± 3	11 ± 4	5 ± 4
6 (*n *= 75)	10 ± 3	10 ± 2	7 ± 2
**Overall mean ± SE**	**12 ± 5**	**6 ± 2**	**4 ± 1**

The data are mean numbers of positive cells (± standard error) per field of view; *n *= mean number of total cells sampled per field of view as assessed by counting the numbers of nuclei stained by DAPI.

### RT-PCR analysis of CCR9

**Figure 3:**
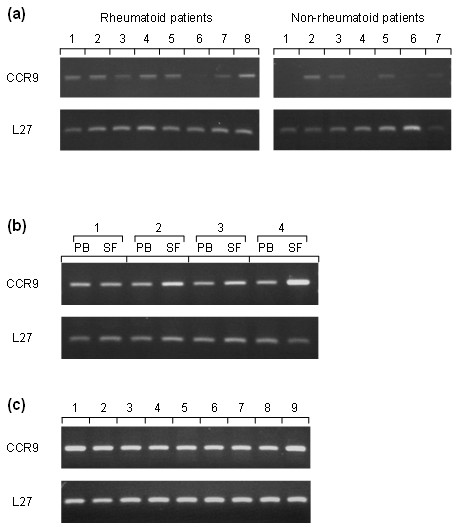
**CCR9 mRNA in synovial tissue and monocytes/macrophages by RT-PCR**. **(a) **RNA isolated from the synovia of rheumatoid arthritis patients (*n *= 8) and controls (*n *= 7) showing the presence of CCR9 mRNA. **(b) **CCR9 mRNA expression by monocytes/macrophages isolated from peripheral blood (PB) and paired synovial fluid (SF) from four RA patients. **(c) **CCR9 mRNA detected in monocytes/macrophages isolated from the PB of normal healthy volunteers. L27 was used for normalisation.

To support the immunohistology data, mRNA expression was analysed in RA and non-RA synovia. PCR primers were run through a BLAST program to ensure gene specificity of the RT-PCR results and to exclude the possibility of cross-hybridization with other genes. CCR9 mRNA was present in the synovia of all eight patients though at a much lower level in Patient 6 (Figure 3a). *CCR9 *was also detected in non-RA samples however only in four out of seven patients. The PCR reactions were normalised with *L27 *(*L27 *ribosomal gene) to allow for comparison between samples.

To further investigate whether macrophages are themselves producing CCR9 and confirm the flow cytometric results, we performed RT-PCR on monocytes/macrophages isolated from PB and SF of four RA patients and PB of nine healthy volunteers. CCR9 mRNA was expressed in isolated RA monocytes/macrophages from both PB and SF (Figure 3b). The message for this receptor was also present in PB monocytes/macrophages from healthy donors (Figure 3c).

### CCL25 expression

**Figure 4:**
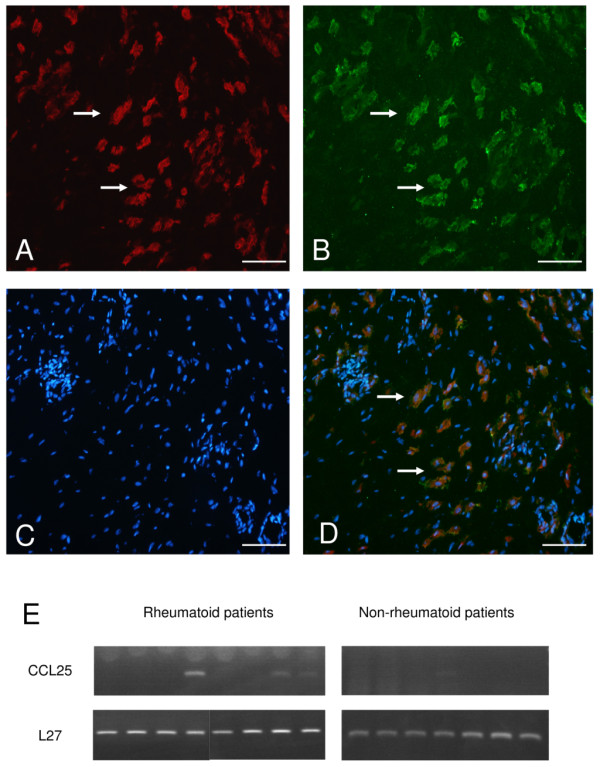
**CCL25 expression in the rheumatoid synovium**. Sections were treated with antibodies to CCL25 and CD68, followed by double label immunofluorescence microscopy. **(a) **Shows staining for CD68 (red) in cells of the synovial sublining. **(b) **Same field of view as A labelled for CCL25 (green). **(c) **Shows staining for nuclei using DAPI (blue). **(d) **Merge of A, B and C showing CD68+CCL25+ cells. Arrows indicate examples of cells where CD68 and CCL25 colocalise. The bar represents 100 μm. E, CCL25 mRNA in synovial tissue by RT-PCR. RNA isolated from the synovia of rheumatoid arthritis patients (*n *= 8) and controls (*n *= 7) showing the presence of CCL25 mRNA. L27 was used for normalisation.

The expression of CCL25 in the synovium was investigated by immunofluorescence microscopy of cryosections. CCL25 was expressed in synovia of all RA (*n *= 8) and non-RA (*n *= 5) patients. The chemokine was observed in cells of the sublining. Double labelling using antibodies to CD14 or CD68 together with CCL25, identified CCL25+ cells as being macrophages (Figure 4a-d). No staining was detected in the isotype controls.

Messenger RNA expression was analysed in RA and non-RA synovia. CCL25 mRNA was present in three out of eight RA samples (Figure 4e). In non-RA synovia *CCL25 *was also detectable however only in one out of seven samples. The PCR reactions were normalised with *L27 *(L27 ribosomal gene). These results indicate that CCL25 message is detectable in synovia although, unlike the protein it was not present in every sample. The lack of exact correlation between mRNA and protein has been shown for other genes [25] and suggests that CCL25 mRNA and protein may be differentially regulated.

### TNFα-stimulated up-regulation of CCR9 on THP-1 cells

**Figure 5:**
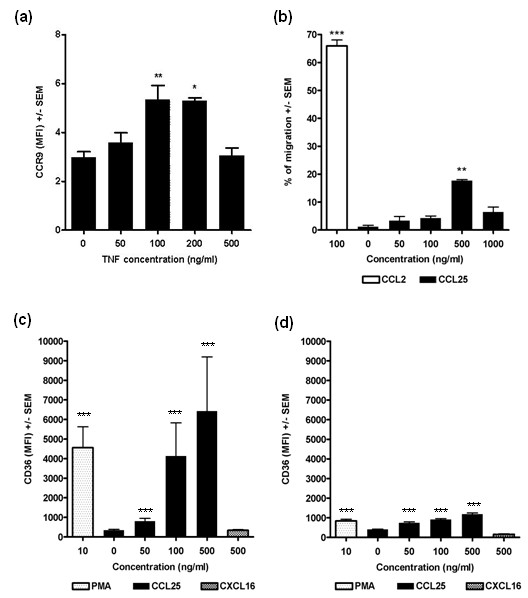
**Regulation and function of CCR9**. **(a) **Up-regulation of CCR9 on THP-1 monocytic cells by TNFα. Cells were cultured for 16 hours in the absence or presence of various concentrations of TNFα, then analysed for CCR9 expression by flow cytometry, *P < 0.05 and **P < 0.01 compared to the absence of TNFα. **(b) **Chemotactic response of peripheral blood monocytes from healthy donors to increasing concentrations of CCL25, CCL2 (MCP-1) as positive control, from 1 representative healthy donor in duplicate.** = *P *< 0.01 compared to control. **(c, d) **CCL25 stimulates CD36 expression by RA (c) and non-RA (d) monocytes. Peripheral blood monocytes were isolated and treated with CCL25, CXCL16 or PMA for three hours, followed by flow cytometry to detect CD36. Data for CCL25 and PMA are means ± standard errors from five RA and non-RA individuals all in triplicate. *** = *P *< 0.0001 compared to control (no CCL25). The rise in CD36 expression in response to CCL25 is greater for RA (c) than healthy (d) monocytes (*P *< 0.01 at 50 ng/ml, *P *< 0.001 at 100 ng/ml and 500 ng/ml CCL25 compared to control). The CXCL16 data represent two RA and two non-RA individuals in triplicate.

To examine the effects of activation on CCR9 expression by monocytes, the monocytic cell line THP-1 was stimulated with TNFα (0 to 500 ng/ml) [23] (Figure 5a). This cytokine elicited an up-regulation of CCR9 expression as shown by flow cytometry (*P *< 0.01). There was a maximum stimulation at 100 ng/ml (*P *< 0.01) and 200 ng/ml (*P *< 0.05) TNFα compared to control.

### Functional aspects of monocyte CCR9

To investigate the functionality of CCR9 chemotaxis experiments were performed. PB monocytes responded to CCL25 (*P *< 0.01) with an optimal response at 500 ng/ml (*P *< 0.01) (Figure 5b). However, chemotactic responses to CCL25 were not observed in all individuals despite showing significant responses to CCL2, the classic monocyte attracting chemokine. Identical results were obtained for the CD14+CD16- and CD14+CD16+ subpopulations (not shown).

To examine if CCL25 affected the differentiation of monocytes, its influence on the levels of the scavenger receptor CD36 was studied. Preliminary experiments using the monocytic cell line U937 showed that CCL25 (0 to 500 ng/ml) induced differentiation of these cells as indicated by a concentration dependent increase of CD36 levels (*P *< 0.0001) (data not shown).

On RA monocytes CCL25 (0 to 500 ng/ml) gave a significant increase in CD36 expression on RA monocytes (*P *< 0.001) (Figure 5c). At 50 ng/ml, 100 ng/ml and 500 ng/ml CCL25, CD36 levels were significantly higher than in the absence of chemokine (*P *< 0.001). PMA (positive control) also gave an increase in CD36 expression (*P *< 0.001) compared to control. In healthy individuals CCL25 also stimulated the expression of CD36 (*P *< 0.001) on monocytes (Figure 5d). There were significant increased levels of CD36 at all concentrations tested compared to the no chemokine control (*P *< 0.001 in each case). PMA also gave a significant stimulation in CD36 levels compared to control (*P *< 0.001).

There was no significant difference in CD36 expression between control RA and healthy monocytes in the absence of CCL25. However, in the presence of CCL25 RA monocytes were more responsive than healthy monocytes (Figure 5c, d). This was shown by the rise in CD36 levels from baseline to 50, 100 and 500 ng/ml CCL25 being significantly greater for RA than healthy monocytes (*P *= 0.007, *P *< 0.001 and *P *< 0.001 respectively). Furthermore, at 100 ng/ml and 500 ng/ml CCL25 RA monocytes displayed marked increases in CD36 expression amounting to 12- and 19-fold respectively compared to no chemokine control, whereas non-RA monocytes gave only 2- and 3-fold increases respectively. Similarly for PMA stimulation, the rise in CD36 expression was greater on RA than healthy monocytes (*P *< 0.001).

CXCR6 was expressed at very low levels on RA and healthy PB monocytes by flow cytometry (MFI <0.46), in agreement with others [30]. Addition of CXCL16 (the ligand for CXCR6) at 50, 100 and 500 ng/ml gave no significant increase in CD36 levels on RA and healthy monocytes for each concentration (Figure 5c, d).

CCL25 also stimulated the expression of alternative macrophage scavenger receptors CD91 and SR-A by RA and healthy monocytes (data not shown).

## Discussion

The results of this study show an increased expression of CCR9 on circulating monocytes in the blood of patients with RA. Not only the number of CCR9 receptors per monocytic cell increases but also the percentage of monocytic cells bearing this receptor increases. Furthermore, in the synovium there is an increase in the number and percentage of macrophages that express CCR9 in RA. The percentage of CCR9+ monocytes in RA synovium (81%) is greater than that in blood (40%), similarly in non-RA the percentage of CCR9+ monocytes in the synovium (66%) is increased compared to that in blood (16%). This suggests there may be a preferential recruitment of monocytes bearing this receptor into the synovium or an up-regulation of the receptor, possibly by cytokines (for example, TNF), once the cells have migrated into the tissue.

Monocytes migrate from the blood into tissues and differentiate into resident macrophages. This occurs via sublining blood vessels in the normal and RA synovium. However, in the latter the number of monocytes recruited and the accumulation of macrophages is markedly increased. When monocytes differentiate into macrophages they up-regulate pattern recognition receptors, such as scavenger receptors and toll-like receptors [31]. Like other scavenger receptors CD36 acts as a phagocytic receptor [32] and was used in the current study as a marker of monocyte to macrophage differentiation [33]. In addition CD36 has been detected on macrophages in the RA joint [34].

A major finding of the current study was the stimulatory effect of CCL25 on monocyte differentiation. The results show that in RA the basal CD36 levels on monocytes are similar to those of normal monocytes. However, upon CCL25 stimulation RA monocytes are significantly more responsive in terms of CD36 expression. This increased responsiveness may be related to the increased abundance of CCR9 found on RA blood monocytes, as shown by the MFI flow cytometry data. In addition, the increased responsiveness was not only CCL25-related since PMA, a known inducer of monocyte differentiation [35], also elicited a greater response in RA monocytes compared to normal. CCL25 appears to function like CXCL4 which is another chemokine reported to stimulate the differentiation of monocytes to macrophages [36]. It is unknown, as yet, whether CCR9 is modulated during monocyte differentiation and differentially expressed by polarized (M1 and M2) macrophages, together with the effect of CCL25 on markers of macrophage polarization.

CCL25 was originally defined as being chemotactic for activated, but not resting, human monocytic THP-1 cells, and activated mouse peritoneal macrophages [23]. This implies the existence of CCR9 on activated monocytes and macrophages, although this was not formally shown experimentally. In order to further this study of Vicari *et al. *[23], we showed that activation of THP-1 cells with TNFα results in increased CCR9 expression. In RA, monocytes in the circulation show evidence of increased activation prior to their entry into the synovium, as well as in the synovium itself [10]. Therefore the increase in the expression of CCR9 may be related to the enhanced activation state of monocytes/macrophages in the RA circulation and synovial tissue. The mechanism behind the up-regulation of CCR9 on monocytes in the blood is unknown but may relate to elevated cytokines, such as TNFα, in the circulation which occurs in RA [37]. Alternatively the increased CCR9 expression on monocytes may be due to changes occurring in the bone marrow which are known to occur in the disease [38-40].

The level of CCR9 expression on blood monocytes in RA and healthy patients was compared with other chemokine receptors using flow cytometry. In terms of MFI and the percentage positive cells, CCR9 expression was similar to CCR5 and CXCR4, although was not as highly expressed as CCR2 and CCR1.

CCR9 is described as an important chemokine receptor in the gut environment where it is constitutively expressed in the majority of CD4+ and CD8+ T lymphocytes of the small intestine [15,17,41,42]. In the circulation it is also expressed by discrete subsets of memory CD4+ and CD8+ lymphocytes expressing the intestinal homing receptor α4β7 [15]. Adoptive transfer models and *in vivo *neutralisation experiments have revealed that CCR9 and CCL25 play important roles in the localisation of effector T cells to the small intestinal mucosa [43,44]. In the present study, CCR9 was not detected in T lymphocytes in the RA and non-RA synovium. This suggests that the CCR9+/α4β7+ subset of T cells are not recruited to the synovium and other chemokine receptors and adhesion molecules are important for T cell migration into this tissue [17,45]. Furthermore, our data support the notion of a tissue-specific address code for T cell recruitment to the synovium that is different from the gut. In inflammatory gut disease, CCR9+ T cells are more abundant in the PB of patients with celiac disease or Crohn's disease [42]. We observed a similar pattern for monocytes in RA patients. However, the percentage of CCR9+ T lymphocytes was shown to be reduced in the small intestine with Crohn's disease [42,43,46] whereas in the present study the percentage of CCR9+ macrophages in RA ST did not reduce compared with normal, but was increased.

CCL25 has been shown to be produced by dendritic and epithelial cells in the normal thymus [23,46,47]. In addition, in the small intestine CCL25 is constitutively expressed by epithelial and endothelial cells [17,23,41]. In inflamed small intestine in Crohn's disease CCL25 expression is in epithelial cells in proximity to lymphocytic infiltrates and is not detectable on endothelial cells [42]. In the present study CCL25 localised to CD14+ and CD68+ cells in the synovial sublining indicating that macrophages are a source of this chemokine. Furthermore the production of CCL25 by synovium is supported by the presence of CCL25 mRNA in RA and non-RA synovial tissue, although levels were low. Our results suggest a positive autocrine loop. Macrophages present in the synovial sublining produce CCL25 leading to the local stimulation of monocyte differentiation to macrophages. Interestingly CD14+ macrophages are likely to represent a recently recruited immature subpopulation of macrophages in the synovial sublining, losing CD14 expression upon maturity [3]. Therefore CCR9 on these CD14+ cells may play a role in the differentiation of recently immigrated monocytes. CCL25 was also present in non-inflamed control synovia and this chemokine stimulated CD36 up-regulation by monocytes from normal healthy donors, although less so compared to RA. Therefore it is possible that CCL25 may enhance the differentiation of monocytes to macrophages under constitutive conditions in the non-inflamed synovium. The effect of CCL25 on monocyte chemotaxis was not consistent; therefore this chemokine may be more effective at influencing differentiation rather than monocyte recruitment.

A very recent *in vivo *paper by Jacobs *et al. *[48] reported the use the K/BxN serum-transfer model of arthritis in mice. CXCR2 was found to be critical for the development of autoantibody-mediated arthritis, and neutrophil recruitment to the joints. This model reflects the effector phase of arthritis rather than the initial adaptive immune response. Other chemokine receptors such as CCR9, CCR1-7, CXCR3, CXCR5 and CX3CR1 were not critical in the model. Many of these receptors have been shown to play a role in arthritis using alternative models that involve an adaptive immune response, probably functioning at that stage. There have been no studies targeting CCR9 in these adaptive immune response models and it would be interesting to perform such a study. An alternative approach to assess the role of CCR9 *in vivo *in RA would be to perform a human study. There is currently a CCR9 antagonist that shows promise in clinical trials for Crohn's disease [49,50], in fact this is one of the few clinical trials targeting chemokine receptors that show hope in the treatment of inflammatory diseases. So a clinical study using this antagonist in human RA would be of interest, also overcoming problems of lack of exact correlation between animal models of RA and human disease.

## Conclusions

Macrophages are held to be an important cell type responsible for the pathogenesis of RA [51]. These cells are major sources of cytokines, such as TNF, chemokines, and degradative enzymes that drive joint inflammation and destruction. Furthermore, it is indicated that they play a role in angiogenesis and antigen presentation. The present study shows the presence of CCR9 on monocytes/macrophges in RA where this receptor stimulates monocyte differentiation. Therefore CCR9 may be an interesting therapeutic target in RA and suggests the use of CCR9 antagonists in the disease.

## Abbreviations

CCL25: C C chemokine ligand 25/TECK; CD14+: cluster of differentiation 14+; CD68+: cluster of differentiation 68+; DAB: diaminobenzidine; MFI: mean fluorescence intensity; PB: peripheral blood; PBS: phosphate buffered saline; PMA: phorbal myristrate acetate; RA: rheumatoid arthritis; RT-PCR: reverse transcription polymerase chain reaction; SF: synovial fluid; THP-1: human acute monocytic leukemia cell line; TNFα: tumour necrosis factor alpha.

## Competing interests

The authors declare that they have no competing interests.

## Authors' contributions

CS carried out the experimental work, analysed the data and wrote the manuscript. AC, HW and OH carried out the experimental work and analysed the data. JW, AF, MS and CB were involved in the design of the study and evaluation of the manuscript. JM designed the study, analysed the data and wrote the manuscript.
